# The Abscopal Effects of Cranial Irradiation Induce Testicular Damage in Mice

**DOI:** 10.3389/fphys.2021.717571

**Published:** 2021-09-30

**Authors:** Ling Guo, Tong-Zhou Qin, Li-Yuan Liu, Pan-Pan Lai, Yi-Zhe Xue, Yun-Tao Jing, Wei Zhang, Wei Li, Jing Li, Gui-Rong Ding

**Affiliations:** ^1^ Department of Radiation Protection Medicine, School of Military Preventive Medicine, Fourth Military Medical University, Xi’an, China; ^2^ Ministry of Education Key Lab of Hazard Assessment and Control in Special Operational Environment, Xi’an, China; ^3^ Department of Histology and Embryology, School of Basic Medical Science, Fourth Military Medical University, Xi’an, China

**Keywords:** cranial irradiation, abscopal effect, testicular damage, apoptosis, sperm quality

## Abstract

To investigate whether the abscopal effects of cranial irradiation (C-irradiation) cause testicular damage in mice, male *C57BL/6* mice (9weeks of age) were randomly divided into a sham irradiation group, a shielded group and a C-irradiation group and administered sham/shielded irradiation or C-irradiation at a dose rate of 2.33Gy/min (5Gy/d for 4 d consecutively). All mice were sacrificed at 4weeks after C-irradiation. We calculated the testis index, observed testicular histology by haematoxylin-eosin (HE) staining and observed testicular ultrastructure by transmission electron microscopy. Western blotting was used to determine the protein levels of Bax, Bcl-2, Cleaved caspase 3, glial cell line-derived neurotrophic factor (GDNF) and stem cell factor (SCF) in the testes of mice. Immunofluorescence staining was performed to detect the expression of Cleaved caspase 3 and 3β hydroxysteroid dehydrogenase (3βHSD), and a TUNEL assay was used to confirm the location of apoptotic cells. The levels of testosterone (T), GDNF and SCF were measured by ELISA. We also evaluated the sperm quality in the cauda epididymides by measuring the sperm count, abnormality, survival rate and apoptosis rate. The results showed that there was no significant difference in testicular histology, ultrastructure or sperm quality between the shielded group and sham group. Compared with the sham/shielded group, the C-irradiation group exhibited a lower testis index and severely damaged testicular histology and ultrastructure at 4weeks after C-irradiation. The levels of apoptosis in the testes increased markedly in the C-irradiation group, especially in spermatogonial stem cells. The levels of serum T and testicular 3βHSD did not obviously differ between the sham group and the C-irradiation group, but the levels of GDNF and SCF in the testes increased in the C-irradiation group, compared with the sham group. In addition, the sperm count and survival rate decreased in the C-irradiation group, while the abnormality and apoptosis rate increased. Under these experimental conditions, the abscopal effects of C-irradiation induced testicular damage with regard to both structure and function and ultimately decreased sperm quality in mice. These findings provide novel insights into prevention and treatment targets for male reproductive damage induced by C-irradiation.

## Introduction

According to recent WHO statistics, head and neck cancer is the seventh most common cancer overall (and the fifth most common cancer in men) worldwide, accounting for an estimated 888,000 new cases in 2018 (Wild et al., 2020). Notably, its incidence is increasing each year, and there is a trend towards a decreasing age at onset; thus, this disease seriously threatens human health. Cranial irradiation (C-irradiation) therapy is one of the major treatment modalities for primary and metastatic head and neck cancer (Siegel et al., 2020; Turnquist et al., 2020). Hypofractionated radiation (single dose >2.5 Gy) is a promising new strategy for radiotherapy due to its higher treatment ratio, shorter total treatment time and lower cost than conventional radiotherapy (single dose=2.0Gy; Azoulay et al., 2017; Isfahanian et al., 2017; Rudat et al., 2017; Vischioni et al., 2019).

Notably, cell and tissue injuries can occur in organs other than the irradiated tumour sites over the course of radiotherapy; such effects are called radiation-induced abscopal effects (RIAEs; Siva et al., 2015; Hu and Shao, 2020). Most previous literature on RIAEs has focused on the regression of nonirradiated metastatic lesions after localised tumour radiotherapy (Ishiyama et al., 2012; Siva et al., 2015; Abuodeh et al., 2016; Seggelen et al., 2020). However, RIAEs also include serious side effects in normal tissues (Aravindan et al., 2014; Tu et al., 2019). Extracranial abscopal effects of C-irradiation are particularly unusual given the brain’s distinctive immune microenvironment (Lin et al., 2019). However, multiple reports have shown that C-irradiation can cause serious abscopal effects in normal peripheral tissues, such as the haemopoietic system, thymus, lungs and spleen (Koturbash et al., 2008; Lei et al., 2015; Cai et al., 2017; Mohye et al., 2017).

A recent study demonstrated that adult survivors experience a greater decline in sexual functioning after C-irradiation therapy at a dose of >22Gy than after C-irradiation therapy at lower doses (Huang et al., 2020). To date, there have been only two reports about the abscopal effects of C-irradiation on male reproduction in animal models, which focused on DNA damage in the germline (Tamminga et al., 2008) and sperm quality impairment (Zhang et al., 2006). Overall, data on the abscopal effects of C-irradiation on distant testes are scarce, and the effects remain poorly understood. To provide a possible target for improving radiation protection and safety, we studied the abscopal effects of C-irradiation in a hypofractionated regime on the structure and function of the testes in adult mice.

## Materials and Methods

### Animals

Healthy adult male C57BL/6 mice [9weeks of age, certificate number: XK (Shaan 2014–002)] were purchased from the Laboratory Animal Center of the Fourth Military Medical University (Xi’an, China) and maintained (four mice per cage) in the animal facility (12-h light/dark cycle; temperature, 20–26°C; and humidity, 45–65%) with free access to food and water. After 1 week of adaptive feeding, the mice were randomly divided into a sham irradiation group and a C-irradiation group (*n*=16 for each group). Notably, to ensure that no radiation leaked through the lead shield and that protection of the shielded ‘bystander’ tissue was complete, we added a shielded irradiation group (shielded group, eight mice). All procedures in this study were approved and conducted following the guidelines of the Animal Welfare Committee of the Fourth Military Medical University (Xi’an, China).

### Procedure of C-Irradiation

For the C-irradiation group, the mice were kept in a conscious state and administered C-irradiation in four hypofractionated doses of X-rays (RAD Source RS 2000 series, Suwanee, United States; working electric current 25mA, working voltage 160kV) 5Gy/d for 4 d consecutively at a dose rate of 2.33Gy/min, which was monitored in real time by a radiation dosimeter (Radcal Accu-Dose, United States). The remainder of each mouse’s body was completely protected by a 2-cm thick lead shield. The dose rate of the testes under the lead shield was 0.01Gy/min, which was equivalent to four thousandths of the cranial dose. For the shielded group, the whole body of each mouse was placed under a 2-cm lead shield and then irradiated in the same way as the C-irradiation group. Besides, the dose rate was 0.01Gy/min and the total does was 0.08Gy. For the sham group, the mice were subjected to the same procedure as the mice in the C-irradiation group except for X-ray irradiation.

### Sample Collection and Testis Index Calculation

The body weight of each mouse was recorded every 3days. All mice were fed for 4weeks after C-irradiation and then euthanized with 1% sodium pentobarbital (50mg/kg). Immediately, the bilateral testes were quickly freed from the surrounding connective tissues and excised after transcranial perfusion with 0.9% sodium chloride. The tissues were rinsed with precooled phosphate-buffered saline (PBS), immediately weighed, snap-frozen in liquid nitrogen and stored at −80°C until analysis. The testis index was calculated using the following formula: bilateral testes weight (g)/body weight (g)×100%.

### Observation of Testicular Histology by HE Staining

After anaesthesia, mice (*n*=2–4 for each group) were fixed *via* cardiac perfusion with 4% paraformaldehyde (PFA, pH=7.3) after transcranial perfusion with 0.9% sodium chloride. The bilateral testes were fixed in Bouin’s fixative solution (Lilai, Chengdu, China) for morphological examination. After fixation for 24h, the fixed testes were routinely trimmed, dehydrated, embedded in paraffin and then serially sectioned on a rotary microtome (RM2135, Leica, Heidelberg, Germany) at a thickness of 4μm. Before staining, the tissue sections were preheated at 60°C for 2h, deparaffinised, rehydrated in graded ethanol and stained with haematoxylin-eosin (HE) according to routine protocols. Then, histological changes were observed with a light microscope (Leica).

### Histological Analysis of Testis

For histological analysis of testis, the diameter of seminiferous tubules and height of seminiferous epithelium were measured using ImageJ software (NIH, MD, United States) from 50 random round or nearly round seminiferous tubules at ×100 magnification for each group, according to the methods in a previous study (Babazadeh and Najafi, 2017; Guo et al., 2019). Briefly, the diameter was calculated as the mean of the major and minor axes of the seminiferous tubules, and the height of the seminiferous epithelium was calculated as follows (average diameter – average inner diameter) of seminiferous tubule/2. In addition, according to the appearance of cells present in the seminiferous tubules, the seminiferous tubules were divided into normal or abnormal (Ibáñez et al., 2017), and the percentage of abnormal seminiferous tubules was counted from 10 random fields at ×100 magnification for each group.

### Observation of Testicular Ultrastructure by Transmission Electron Microscopy

After anaesthesia, mouse testes (*n*=2 for each group) were separated, trimmed to 1mm×1mm×1mm samples, fixed in 3% glutaraldehyde and 1% osmic acid, dehydrated in a graded series of acetone (30, 50, 70, 80, 90, 95 and 100%) and then embedded in Araldite. Ultrathin slices (50nm thick) were double-stained with saturated uranyl acetate and lead citrate. A transmission electron microscope (JEM-1400FLASH; JEOL Ltd., Tokyo, Japan) was used to observe the ultrastructure of seminiferous tubules. The testicular ultrastructure observed in this study included mainly Sertoli cells, Leydig cells, spermatogonia, spermatocytes and spermatids.

### Western Blotting

Total testicular protein (*n*=4 for each group) was extracted and quantified as described previously. Equal amounts of testis samples (30μg) were subjected to 10–12% Bis-Tris gel electrophoresis and transferred to polyvinylidene fluoride immunoblot membranes (0.22μm). The membranes were blocked with 5% non-fat milk for 2h at room temperature and probed with primary antibodies overnight at 4°C. Primary antibodies against β-actin (20536-1-AP, 1:5000), Bcl-2 (12789-1-AP, 1:2000) and Bax (50599-2-Ig, 1:3000) were obtained from Proteintech (Wuhan, China); primary antibodies against SCF (21670–1, 1:300) were obtained from SAB (MD, United States); and primary antibodies against Cleaved caspase 3 (ab214430, 1:4000) and GDNF (ab176564, 1:2000) were obtained from Abcam (MA, United States). The following morning, the membranes were incubated with species-matched horseradish peroxidase (HRP)-conjugated secondary antibodies (1:5000, CWBIO, Beijing, China) for 2h at room temperature and then incubated with chemiluminescent HRP substrate to visualise the bands. Quantity One 4.62 software (Bio-Rad, CA, United States) was used to analyse the optical density of each target band. To normalise the protein levels, β-actin was used as a loading control.

### Immunofluorescence Staining and TUNEL Assay

After initial deparaffinization and rehydration, testis sections were processed by antigen retrieval using citrate buffer in a high-power microwave oven, treated with 3% bovine serum albumin for 30min and incubated with a rabbit monoclonal Cleaved caspase 3 antibody (9664S, 1:200, CST, MA, United States) and a rabbit polyclonal 3β hydroxysteroid dehydrogenase (3βHSD) antibody (DF6639, 1:150, Affinity Biosciences, OH, United States) at 4°C overnight. Next day, the sections were subsequently treated with a FITC-conjugated goat anti-rabbit antibody (ab6717, 1:1000, Abcam, MA, United States). Testicular cell apoptosis was assessed by terminal deoxynucleotidyl transferase (TdT) enzymaticated dUTP nick end labelling (TUNEL) assay using an *in situ* Cell Death Detection Kit (Roche, Basel, Switzerland). Briefly, after initial deparaffinization and antigen recovery, the section was permeabilised with Triton X-100 (ST795, Beyotime, Shanghai, China), followed by 30μl TUNEL reaction mixture for 60min at 37°C. Negative controls were performed without the enzyme TdT. Finally, 10 random fields for each group were chosen at random for analysis using a fluorescence microscope (Leica), and the average fluorescence intensity was calculated using ImageJ software.

### Detection of Testicular Secretory Function by ELISA

Blood samples were taken from the heart and centrifuged at 3000rpm for 15min at 4°C to obtain serum, which was stored at −80°C and used for detection of the secretory function of Leydig cells. The levels of serum testosterone (T; *n*=7 for each group), secreted by Leydig cells, were determined with an ELISA kit (Sinoukbio, Beijing, China) according to the manufacturers’ instructions. In addition, testis tissue (approximately 100mg; *n*=5 for each group) was lysed with PBS and homogenised to extract total proteins in a homogenisation device (Leica) under precooled conditions. After that, the levels of GDNF and SCF in the testis were measured with the ELISA kits (Elabscience, Wuhan, China) according to the manufacturers’ instructions.

### Detection of Sperm Quality

The cauda epididymides of each mouse were dissected out carefully, gently cut, collected in a 12-well plate containing 1mL of sperm culture solution (Millipore, MA, United States) and then incubated at 37°C for 30min. The sperm suspension was collected and filtered through a nylon mesh with a 38-μm pore diameter to remove tissue fragments and then used to record and calculate sperm count and abnormality according to the methods in a previous study (Guo et al., 2019). The types of abnormal sperm morphology observed in this study mainly included the folded-tail, hookless, amorphous, double-head and double-tail phenotypes according to a previous study (Chen et al., 2019). In addition, a FITC annexin V apoptosis detection kit Ι (BD Pharmingen, CA, United States) was applied to quantify the survival rate and apoptosis rate of sperm. Briefly, sperm samples prepared as described above were supplemented with 1mL of 1× annexin V binding buffer. Subsequently, the samples were washed and incubated in annexin V-FITC and propidium iodide (PI) at 37°C for 5min in the dark and then analysed by flow cytometry (FCM; XL-MCL, Beckman Coulter, CA, United States) following the manufacturer’s instructions. Four or five sperm samples were used for each group, and 15,000 sperm were analysed for each sample.

### Statistical Analysis

All measurement data are expressed as the mean and standard deviation (mean±SD) and were analysed with SPSS 20.0 statistical software (SPSS Inc., Chicago, IL, United States). For statistical analysis, two-way ANOVA with repeated measures was used to analyse the body weights of mice, one-way ANOVA followed by Tukey’s multiple comparisons test was used to compare three groups and a two-tailed student’s *t*-test was used to compare two groups for parametric data (data that met the normality and equal variance assumptions). All subjective analyses were performed by individuals blinded to the exposure group. All graphs were generated using GraphPad Prism 8.0 software (San Diego, CA, United States), and the results were considered statistically significant at *p*<0.05.

## Results

### The Abscopal Effects of C-Irradiation Damage Testicular Histology

**Figure 1:**
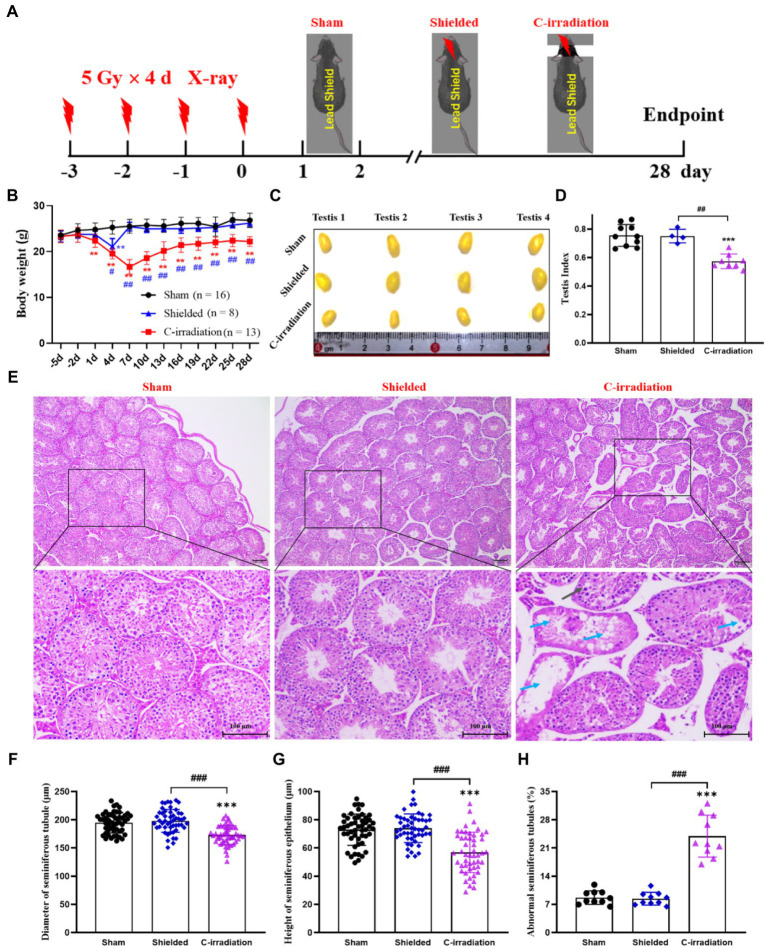
The abscopal effects of C-irradiation damage testicular histology. **(A)** Time schedule of irradiation for mice. **(B)** Body weight of mice during experiments. **(C)** Testis volume, *n*=4 for each group. **(D)** Testis index, *n*=10 for the sham group. *n*=4 for the shielded group and *n*=8 for the C-irradiation group. **(E)** HE staining of testes, *n*=4 for the sham and C-irradiation groups, *n*=2 for the shielded group, bar=100μm. Vacuolation of seminiferous tubules (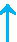) and degeneration and necrosis of various spermatogenic cells (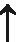). **(F**–**G)** Diameter of seminiferous tubules and height of seminiferous epithelium calculated randomly from 50 round or nearly round cross-sections of the seminiferous tubules (long axis: short axis<1.2:1) for each group. **(H)** Percentage of abnormal seminiferous tubules calculated from 10 random fields for each group. The values are expressed as the mean±SD and analysed by two-way ANOVA for body weight and one-way ANOVA with Tukey’s test for three-group comparisons. ^**^ *p*<0.01; ^***^ *p*<0.001 vs. sham group. ^##^ *p*<0.01; ^###^ *p*<0.001 vs. shielded group.

Figure 1A shows the time schedule of C-irradiation used for the mice. Day 3 to day 0 was the irradiation time. During the whole experiment, the body weights of the mice in the shielded group decreased only on day 4 (Figure 1B, *p*<0.01) and then immediately returned to the levels of the mice in the sham group, and mice in the sham and shielded groups were in good general body conditions. However, from the end of the first day of C-irradiation, mice in the C-irradiation group exhibited evident appetite loss, activity reduction and body weight loss, compared with the mice in the sham and shielded groups (Figure 1B, *p* <0.01), and three mice died due to worsening health status during the first week after C-irradiation. Until 4weeks after C-irradiation, the body weight of mice in the C-irradiation group still lagged significantly (*p*<0.01).

In terms of the reproductive system of male mice, the testicular volume and testis index were significantly lower in the C-irradiation group than in the sham and shielded groups (Figures 1C,D; *p*<0.001), but there was no significant difference between the latter two groups (*p*>0.05). HE staining showed that the testes in the C-irradiation group had obvious pathological changes, such as vacuolation of seminiferous tubules, degeneration and necrosis of spermatogenic cells (Figure 1E). In addition, the diameter of the seminiferous tubules and height of the seminiferous epithelium were significantly lower, and the percentage of abnormal seminiferous tubules was higher in the C-irradiation group than in the sham and shielded groups (Figures 1F,H; *p*<0.001).

Interestingly, there were no significant differences in the organ index for other peripheral organs (heart, liver, spleen, lungs, kidneys and thymus) among the three groups (Figure S1; *p*>0.05). HE staining also showed that compared with the sham group, the histological structures of the other important peripheral organs had no change in the shielded group and no or only slight pathological changes in the C-irradiation group (Figures S2–7). All the above results indicated that the protection of the lead shield was extremely effective, and C-irradiation did not cause obvious scattering to the peripheral organs. Compared with other peripheral organs, testicular tissue was the most sensitive to the abscopal effects of C-irradiation, which could severely damage testicular histology.

### The Abscopal Effects of C-Irradiation Damage the Testicular Ultrastructure

**Figure 2:**
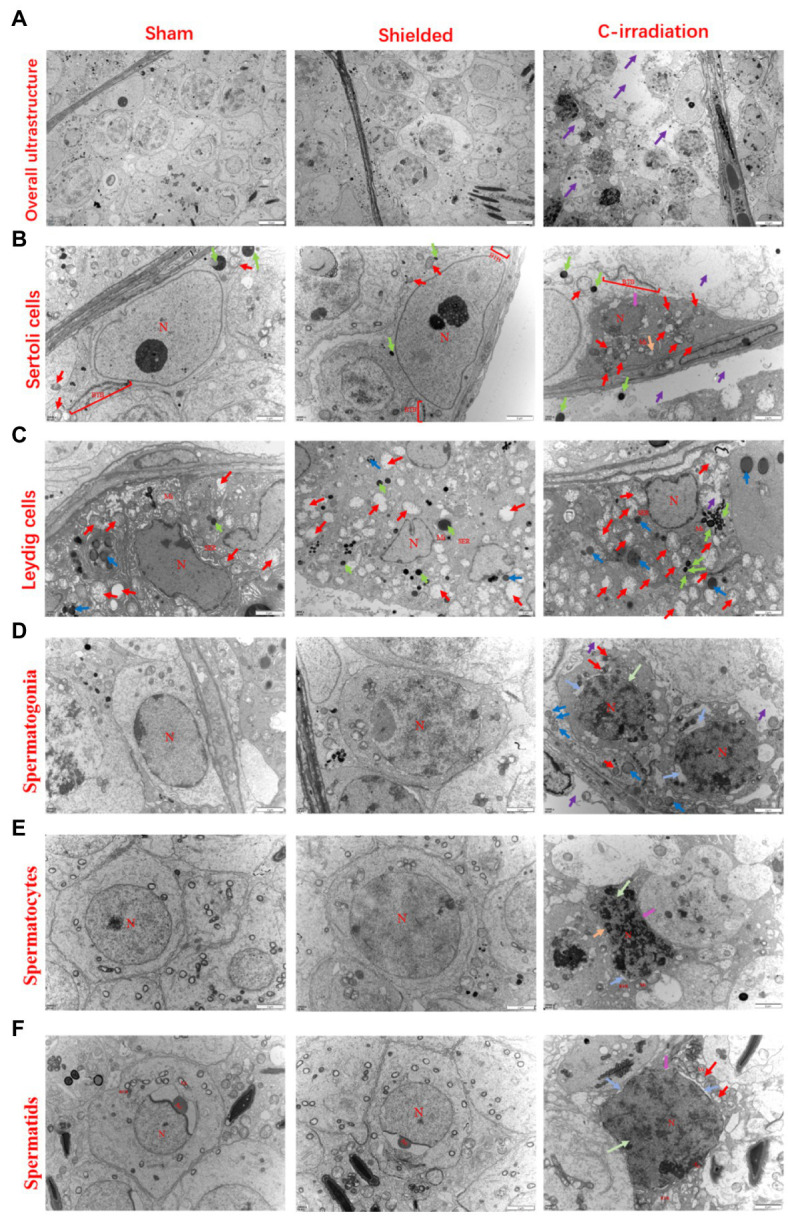
The abscopal effects of C-irradiation damage the testicular ultrastructure. **(A)** Overall ultrastructure of seminiferous tubules. Bar=10μm. **(B**–**F)** Ultrastructure of Sertoli cells, Leydig cells, spermatogonia, spermatocytes and spermatids. Bar=2μm, *n*=2 for each group. N, nucleus; Mi, mitochondrion; RER, rough endoplasmic reticulum; BTB, blood-testis barrier; and Ac, acrosome. Lipid droplets 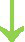, mitochondrial swelling 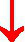, secondary lysosomes 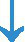, RER dilatation 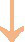, loss of intracytoplasmic solutes 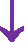, widened perinuclear gap 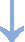 and suspected apoptosis 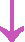.

For the sham and shielded groups, the overall ultrastructure of seminiferous tubules was normal and intact (Figure 2A), and the spermatogenic cells in various growth cycles were closely arranged with clear cell structures, large round or oval nuclei, smooth and clear cell membranes and compact chromatin. The number of organelles in the cytoplasm was normal, and there were abundant mitochondria (Figures 2D–F). The intercellular bridge between the spermatogenic cells and the Sertoli cell junction complex, also called the blood-testis barrier (BTB), was complete (Figure 2B). For the C-irradiation group (Figures 2A–F), the overall ultrastructure of seminiferous tubules was severely damaged, the spermatogenic cell membrane was unclear at all levels, the perinuclear space was expanded, the nuclear membrane was dissolved, the mitochondria showed obvious swelling, cavitation was observed and the endoplasmic reticulum was dilated. Apoptosis and autophagy were suspected to be underway in testicular cells. In addition, the integrity of the BTB was disrupted. All these results suggested that the lead shield had excellent protective effects on the tissues outside the cranial region and that the abscopal effects of C-irradiation severely damaged the testicular ultrastructure.

### The Abscopal Effects of C-Irradiation Increase Testicular Cell Apoptosis

**Figure 3:**
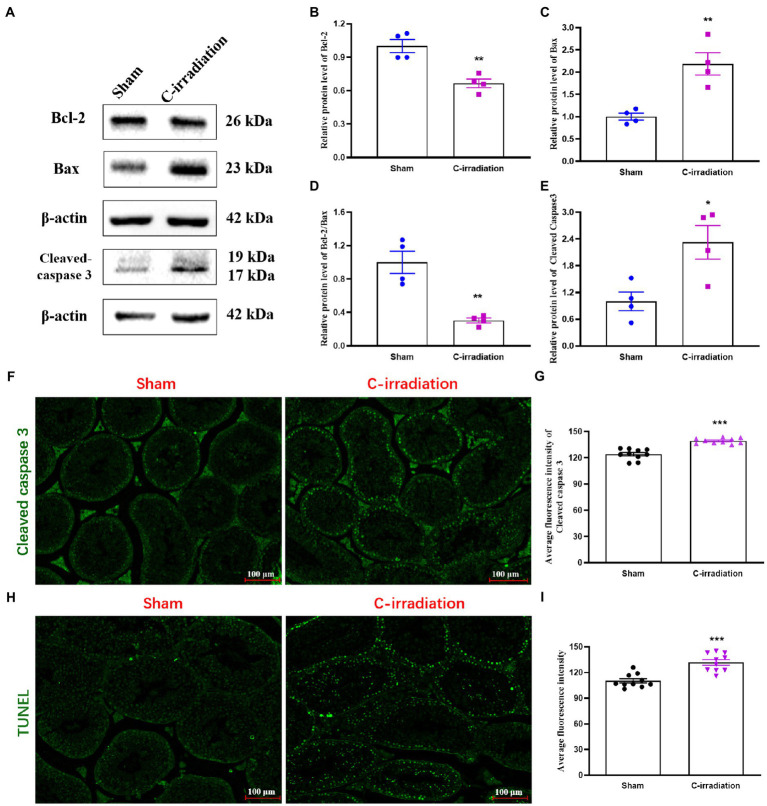
The abscopal effects of C-irradiation increase testicular cell apoptosis. **(A)** Typical immunoblots of apoptosis-related proteins. The first three bands were from the same membrane and the last two bands were from another membrane. **(B**–**E)** Relative protein level of Bcl-2, Bax, Bcl-2/Bax and Cleaved caspase 3 detected by Western blotting, *n*=4 for each group. **(F**–**G)** Immunofluorescence of Cleaved caspase 3 and average fluorescence intensity from 10 random fields for each group. Bar=100μm. **(H**–**I)** TUNEL staining and average fluorescence intensity from 10 random fields for each group. Bar=100μm. The values are expressed as the mean±SD and analysed by two-tailed unpaired student’s *t*-tests. ^*^ *p*<0.05, ^**^ *p*<0.01 and ^***^ *p*<0.001 vs. the sham group.

Western blotting detection of apoptosis-related proteins (Figure 3A) showed that compared with the sham group, the C-irradiation group exhibited significantly lower relative protein level of Bcl-2 (Figure 3B; *p* <0.01), significantly higher relative protein level of Bax (Figure 3C; *p* <0.01), significantly lower Bcl-2/Bax ratio (Figure 3D; *p* <0.05) and significantly higher relative protein level of Cleaved caspase 3 (Figure 3E; *p* <0.05). Immunofluorescence staining of Cleaved caspase 3 showed that the number of Cleaved caspase 3-positive cells increased and that these cells distributed in the outermost seminiferous tubules at 4weeks after C-irradiation (Figures 3F–G; *p* <0.001). In addition, TUNEL staining of testis sections revealed that apoptosis of testicular cells increased obviously in the C-irradiation group compared with sham group (Figures 3H–I; *p* <0.001), and the apoptotic testicular cells also located in the outermost seminiferous tubules. These results are consistent with the above results of Western blotting, suggesting that the abscopal effects of C-irradiation increase testicular cell apoptosis and more in spermatogonial stem cells (SSCs).

### The Abscopal Effects of C-Irradiation Alter the Secretory Functions of the Testes

**Figure 4:**
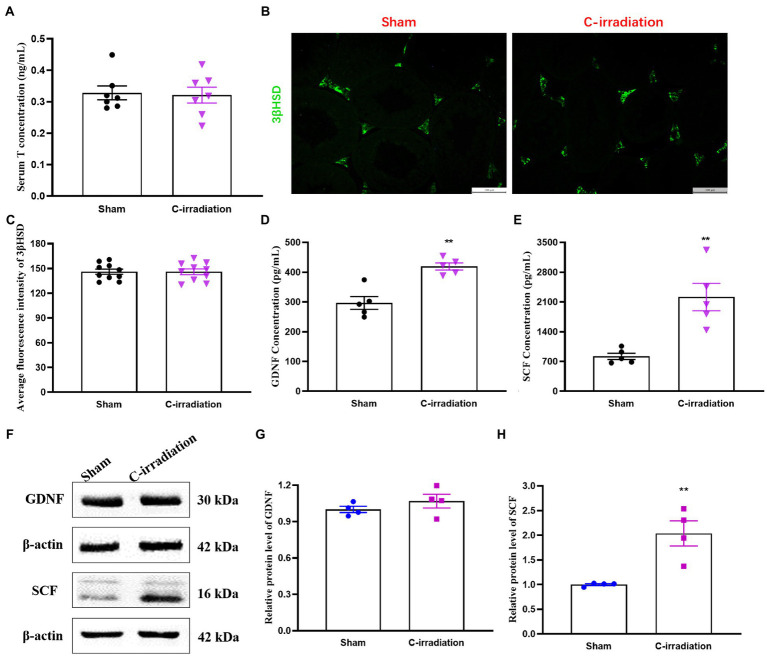
The abscopal effects of C-irradiation affect the secretory functions of the testis. **(A)** Serum T concentrations secreted by Leydig cells, *n*=7 for each group. **(B**–**C)** Immunofluorescence of 3βHSD in testis and average fluorescence intensity from 10 random fields for each group. Bar=100μm. **(D–E)** GDNF and SCF concentrations secreted by Sertoli cells and detected by ELISA, *n*=5 for each group. **(F)** Typical immunoblots relating to the secretory functions of Sertoli cells. The first two bands were from the same membrane and the last two bands were from another membrane. **(G**–**H)** Relative protein level of GDNF and SCF detected by Western blotting, *n*=4 for each group. The values are expressed as the mean±SD and analysed by two-tailed unpaired student’s *t*-tests. ^**^ *p*<0.01 vs. sham group.

The ELISA results showed that the serum T concentration secreted by Leydig cells did not differ between the sham group and the C-irradiation group at 4weeks after C-irradiation (Figure 4A; *p* >0.05). Besides, 3βHSD, a Leydig cell specific marker, plays an important role in the synthesis of steroid hormones (Yang et al., 2017). To explore the abscopal effects of C-irradiation on steroidogenic capacity of Leydig cells, the 3βHSD immunoreactivity in Leydig cells was detected by the immunofluorescence staining in testicular paraffin sections (Figure 4B). The results showed that the level of 3βHSD in testis did not differ between the sham group and the C-irradiation group at 4weeks after C-irradiation (Figure 4C; *p* >0.05), which suggested that the abscopal effects of C-irradiation do not affect the steroidogenic capacity of Leydig cells. However, the concentrations of GDNF and SCF secreted by Sertoli cells were significantly higher in the C-irradiation group at 4weeks after C-irradiation than in the sham group (Figures 4D,E; *p* <0.01). In addition, the results of Western blotting (Figure 4F) showed that compared with the sham group, the relative protein level of GDNF in the C-irradiation group showed an upward trend but not statistically significant (Figure 4G; *p* >0.05), while the relative protein level of SCF was higher at 4weeks after C-irradiation than of the sham group (Figure 4H; *p* <0.01), which was consistent with the results of ELISA. All the results suggest that the abscopal effects of C-irradiation enhance the secretory functions of Sertoli cells at 4weeks after C-irradiation but do not affect the secretory functions of Leydig cells, which may be related to negative feedback of damage repair during this period.

### The Abscopal Effects of C-Irradiation Decrease Sperm Quality

**Figure 5:**
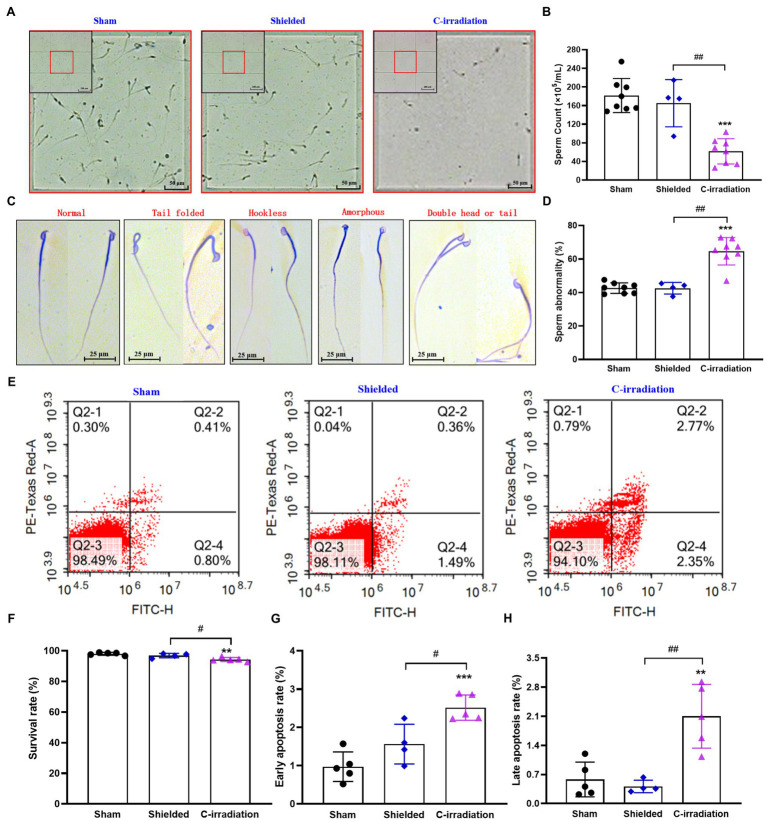
The abscopal effects of C-irradiation decrease the sperm quality of the cauda epididymis. **(A)** Representative pictures of sperm count. Bar=200μm for the upper-left corner and bar=50μm for the magnification with red border. **(B)** Analysis of sperm count. n=8 for the sham and C-irradiation groups and *n*=4 for the shielded group. **(C)** Typical types of abnormal sperm morphology, including the folded-tail, hookless, amorphous, double-head and double-tail phenotypes. Bar=25μm. **(D)** Sperm abnormality. *n*=8 for the sham and C-irradiation groups and *n*=4 for the shielded group. **(E)** Representative pictures of sperm apoptosis detected by FCM. **(F**–**H)** Survival rate, early apoptosis rate and late apoptosis rate of sperm for analysis as noted in **(E)**. *n*=5 for the sham and C-irradiation groups and *n*=4 for the shielded group. The values are expressed as the mean±SD and analysed by one-way ANOVA with Tukey’s test. ^**^ *p*<0.01; ^***^ *p*<0.001 vs. sham group. ^#^ *p*<0.05; ^##^ *p*<0.01 vs. shielded group.

The above results suggest that the testes are the most sensitive target organs to the abscopal effects of C-irradiation. To further clarify the abscopal effects of C-irradiation on testicular function in mice, we detected changes in sperm quality of the cauda epididymis at 4weeks after C-irradiation, including sperm count, abnormality, survival rate and early and late apoptosis rate. Compared with the sham group and shielded group, the C-irradiation group exhibited marked decreases in sperm count (Figures 5A,B; *p* <0.01 or 0.001). Typical types of abnormal sperm morphology observed in this study are shown in Figure 5C, and sperm abnormalities increased obviously in the C-irradiation group (Figure 5D; *p* <0.01 or 0.001). Typical FCM pictures are shown in Figure 5E, where the quadrants represent dead sperm (PI^+^/FITC^−^, upper-left quadrant), late apoptosis sperm (PI^+^/FITC^+^, upper-right quadrant), surviving sperm (PI^−^/FITC^−^, lower-left quadrant) and early apoptosis sperm (PI^−^/FITC^+^, lower-right quadrant). The survival rate of sperm decreased (Figure 5F; *p* <0.05 or 0.01), and the early apoptosis rate and late apoptosis rate of sperm increased significantly at 4weeks after C-irradiation (Figures 5G,H; *p* <0.05 or 0.01 or 0.001). There were no significant changes in any of the above indexes relating to sperm quality in the shielded group, which again indicated that the lead shield had an excellent protective effect and that damage to the testes indeed arose from the abscopal effects of C-irradiation. The above results suggest that the abscopal effects of C-irradiation can damage testicular function and ultimately decrease sperm quality in mice.

## Discussion

With the progress of treatment technology, the survival rates of cancer patients treated with cranial irradiation significantly increased (Xu et al., 2018). Impaired fertility has been recognised as an important quality of life concern for cancer survivors of childbearing age (Muñoz et al., 2016). Thus, protection of the reproductive potential of these patients against C-irradiation damage is important. To our knowledge, this study demonstrates, for the first time, that C-irradiation induces abscopal effects to cause distal testicular damage with regard to both structure and function.

Currently, a hypofractionated dose is being carried out as a new radiotherapy strategy. Thus, 5Gy×4 d C-irradiation was used to explore the damage effect of testicular tissue under shielding in the study. It is possible that X-rays are reflected while passing through tissue, resulting in a small ‘scatter’ dose in the protected tissue. However, a previous study demonstrated that abscopal effects are not the result of insufficient shielding or radiation scattering (Koturbash et al., 2008). Likewise, we administered whole-body shielded irradiation to mice and found that there were no obvious changes in the histological structures of many peripheral organs (Figure S2–7). These results suggested that the protection of the lead shield was extremely effective and that the C-irradiation did not cause obvious scattering to the peripheral organs. We also observed the organ index values and histological structures of important peripheral organs and found that only the testis index decreased and the histological structures of the testis were significantly damaged in the C-irradiation group. All of the above results suggest that the testes are the most sensitive target organs to RIAEs and that the testicular damage in the C-irradiated mice resulted from RIAEs rather than the effects of scattered C-irradiation.

Innumerable studies have proven that the testis is highly sensitive to ionising and nonionizing radiation, which could directly induce testicular cell apoptosis in animals (Said et al., 2019; Rakici et al., 2020). Furthermore, SSCs are highly radiosensitive in spermatogenic populations (Marjault and Allemand, 2016; Qi et al., 2019). However, the sensitivity of spermatogenic populations to RIAEs is unclear. Previous studies have demonstrated that RIAEs can initiate apoptosis in distant tissues (Koturbash et al., 2008; He et al., 2020). In addition, Zhang et al. reported that fractionated irradiation (X-ray, 8Gy×3 d) of the right thorax damaged the ultrastructure of the BTB and increased apoptotic spermatogonia cells, which located at the outermost layer of the seminiferous epithelium of the testis (Zhang et al., 2019). The results are consistent with our findings, indicating SSCs are highly sensitive to RIAE.

The mechanism of testicular cell apoptosis directly induced by ionising radiation is mostly mediated by a p53-dependent Bax-caspase-3-mediated pathway (Shahin et al., 2018; He et al., 2020). Since the testes of mice in the C-irradiation group are not directly exposed to ionising radiation, we speculate that the mechanism of testicular cell apoptosis induced by C-irradiation is different from that induced by direct radiation. Recently, it was reported that abnormal levels of hormones secreted by the hypothalamus-pituitary gland could induce the apoptosis of testicular spermatogenic cells (Chimento et al., 2014). In our ongoing study, we found that the levels of gonadotropin-releasing hormone (GnRH) secreted by hypothalamus, luteinizing hormone (LH) and follicle stimulating hormone (FSH) secreted by pituitary increased significantly at 4weeks after C-irradiation compared with sham group (data not shown). Probably, the testicular cell apoptosis induced by C-irradiation was caused by the abnormal secretory function of the hypothalamus and pituitary gland, and we are trying to get more evident to verify this speculation. Besides, it was reported that the PI3K/Akt pathway, a key regulator of apoptosis, played an important role in testicular damage (Huang et al., 2019; Kucukler et al., 2020; Wang et al., 2021). In addition, SCF and its receptor C-kit are upstream regulators of the PI3K/Akt pathway (Guan et al., 2020). Considering the protein level of SCF in testis increased obviously after C-irradiation compared with sham group, we speculate that another mechanism of testicular apoptosis in this study may be related to the regulation of the SCF/C-kit–PI3K/Akt pathway.

Although testicular histopathology is often considered the gold standard for the nonclinical assessment of testicular damage, male fertility also requires intact testicular function (Kenney et al., 2012; Dere et al., 2013), which depends mostly on the secretory functions of testicular somatic cells (Sertoli cells and Leydig cells; Xiong et al., 2020). T regulated by 3βHSD (Li et al., 2018), synthesised and released by Leydig cells is necessary for both spermatogenesis and the function of Sertoli cells, which secrete proteins necessary for the proliferation and self-renewal of SSCs (Zhang et al., 2006, 2015). In a previous study, 6Gy of C-irradiation with 4 MV of nominal photon energy and a dose rate of 2.3Gy/min induced late-onset T deficiency at 20weeks in juvenile female rats (Xu et al., 2020). However, the levels of serum T and testicular 3βHSD were not altered at 4weeks after 20Gy of C-irradiation in this study. Considering the increase of upstream hormones (GnRH, FSH and LH), we speculated that it is related to negative feedback of damage repair during this period.

Spermatogenic cells are supported by surrounding Sertoli cells, which produce the factors and microenvironment required for each stage of spermatogenic cell development (Walker, 2021; You et al., 2021). The factors include GDNF, which promotes the proliferation and self-renewal of SSCs, and SCF, which encourages the differentiation of SSCs (Guo et al., 2019). The concentrations of GDNF and SCF increased at 4weeks after 20Gy of C-irradiation. We hypothesise that these changes are related to negative feedback regulation of testicular damage repair at 4weeks after C-irradiation, which requires further research.

Spermatogenesis, the primary testicular function, is a complex morphological change of germ cell differentiation that involves self-renewal and differentiation of spermatogonia, meiosis of spermatocytes and spermiogenesis (Huang et al., 2021). Alteration of any stage of spermiogenesis will damage sperm quality and ultimately impact male fertility. The count, survival rate and morphology of sperm are key elements affecting fertility and function as sensitive indexes for evaluating the effects of physical and chemical factors on sperm quality. A previous study (Tamminga et al., 2008) showed that the mature sperm quality of rats decreased on day 7 after X-ray C-irradiation (10Gy×2 d, 3Gy/min). Our results demonstrated that the abscopal effects of hypofractionated C-irradiation decreased the sperm quality of mice, consistent with the findings of Zhang et al.’s (2006) study. That study reported that sperm quality decreased on the 35th day after administration of 2Gy of ^12^C^6+^ ion or ^60^Co γ-ray C-irradiation to mice. Notably, the sperm count in the C-irradiation group decreased drastically. However, in all groups, survival rates were above 93%, and total apoptosis rates were below 5%, indicating that the abscopal effects of C-irradiation mainly impaired spermatogenesis (rather than directly affecting mature sperm) and further reduced sperm quality. Such effects may explain the clinical conditions of temporary infertility and permanent sterility after C-irradiation treatment (Muñoz et al., 2016; Huang et al., 2020; Verbruggen et al., 2021).

The abscopal effects of C-irradiation are dynamic processes mediated by multiple factors, multiple pathways and multiple mechanisms, and they are not mutually exclusive. A clinical study reported that C-irradiation at doses of >22Gy led to gonadotropin deficiency (Haavisto et al., 2016). The hypothalamus-pituitary-gonad axis regulates spermatogenesis in mammals, and the hypothalamus and pituitary are inevitably exposed to radiation during C-irradiation therapy, which may be related to testicular damage resulting from the abscopal effects of C-irradiation. Notably, new technologies, such as gene expression profiling and proteomics, may contribute to elucidation of the mechanism and identification of the molecules involved in testicular damage induced by C-irradiation, which are the focuses of our ongoing research.

## Conclusion

Taken together, the findings of this study indicate that the abscopal effects of C-irradiation can induce testicular damage with regard to both structure and function and ultimately decrease sperm quality in mice. These findings may have important implications for the development of strategies to improve safety and prevent radiotherapy-related reproductive damage.

## Data Availability Statement

The original contributions presented in the study are included in the article/Supplementary Material, and further inquiries can be directed to the corresponding author.

## Ethics Statement

The animal study was reviewed and approved by the Animal Welfare Committee of Fourth Military Medical University.

## Author Contributions

G-RD and LG designed the research. LG, T-ZQ, L-YL, P-PL and Y-ZX performed the research. WZ and WL analysed the data. LG and T-ZQ wrote the manuscript. WL, JL and G-RD revised the manuscript. All authors approved the final manuscript for submission.

## Funding

This work has been carried out with financial support from the Fund of National Natural Science Foundation of China (grant number: 31770905).

## Conflict of Interest

The authors declare that this research was conducted in the absence of any commercial or financial relationships that could be construed as a potential conflict of interest.

## Publisher’s Note

All claims expressed in this article are solely those of the authors and do not necessarily represent those of their affiliated organizations, or those of the publisher, the editors and the reviewers. Any product that may be evaluated in this article, or claim that may be made by its manufacturer, is not guaranteed or endorsed by the publisher.
