# Supplementary material for: The Abscopal Effects of Cranial Irradiation Induce Testicular Damage in Mice
Source: Front Physiol. 2021 Sep 30;12:717571. doi: 10.3389/fphys.2021.717571 (PMC8637864; doi:10.3389/fphys.2021.717571)
Supplement: Supplementary file 1 [file Data_Sheet_1.docx]

Supplementary Material

# Supplementary Figures

**Figure S1.** The abscopal effects of C-irradiation on the organs index of peripheral vital organs in mice. (A) Heart index. (B) Liver index. (C) Spleen index. (D) Lung index. (E) Kidney index. (F) Thymus index. n = 6 for the Sham and C-irradiation groups, n = 4 for the Shielded group. The values are expressed as mean ± SD and analysed by one-way ANOVA with Tukey's test for three groups comparisons.


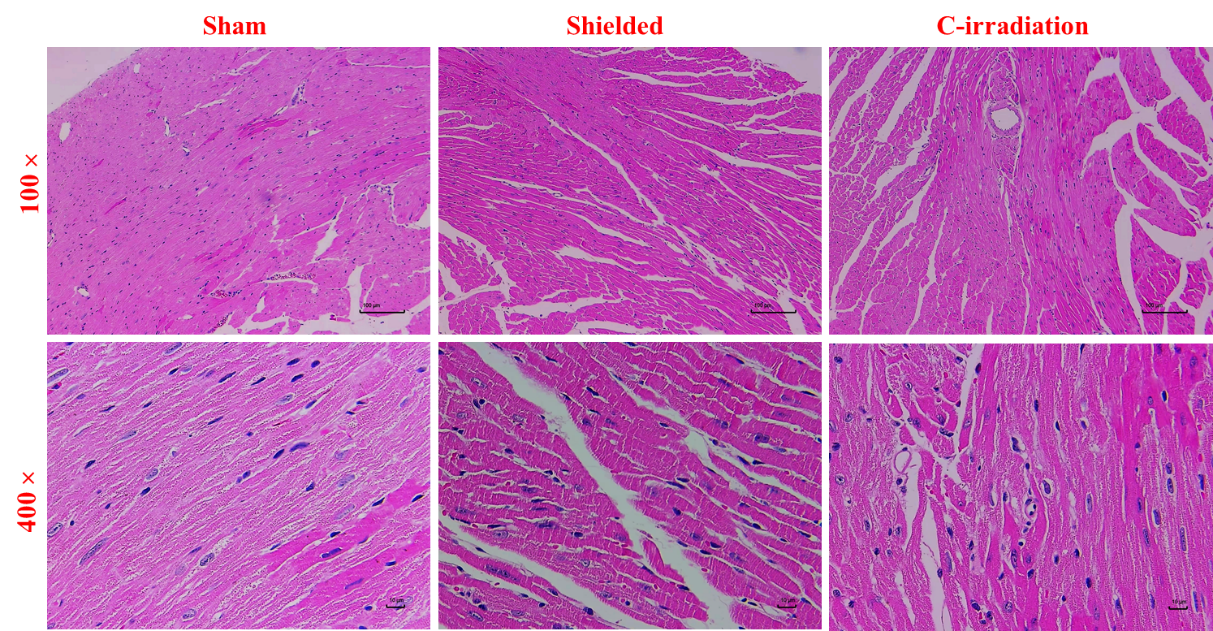


**Figure S2.** The abscopal effects of C-irradiation on the heart of mice, HE staining, bar = 10 μm for 100 × magnification and 100 μm for 400 × magnification.


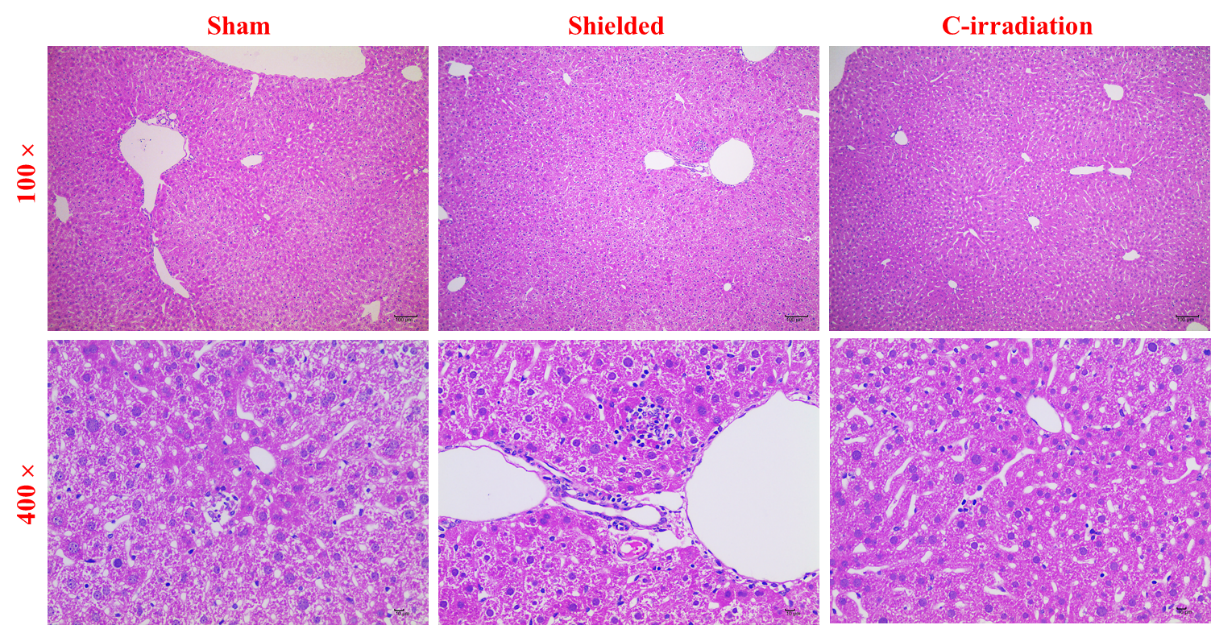


**Figure S3.** The abscopal effects of C-irradiation on the liver of mice, HE staining, bar = 10 μm for 100 × magnification and 100 μm for 400 × magnification.


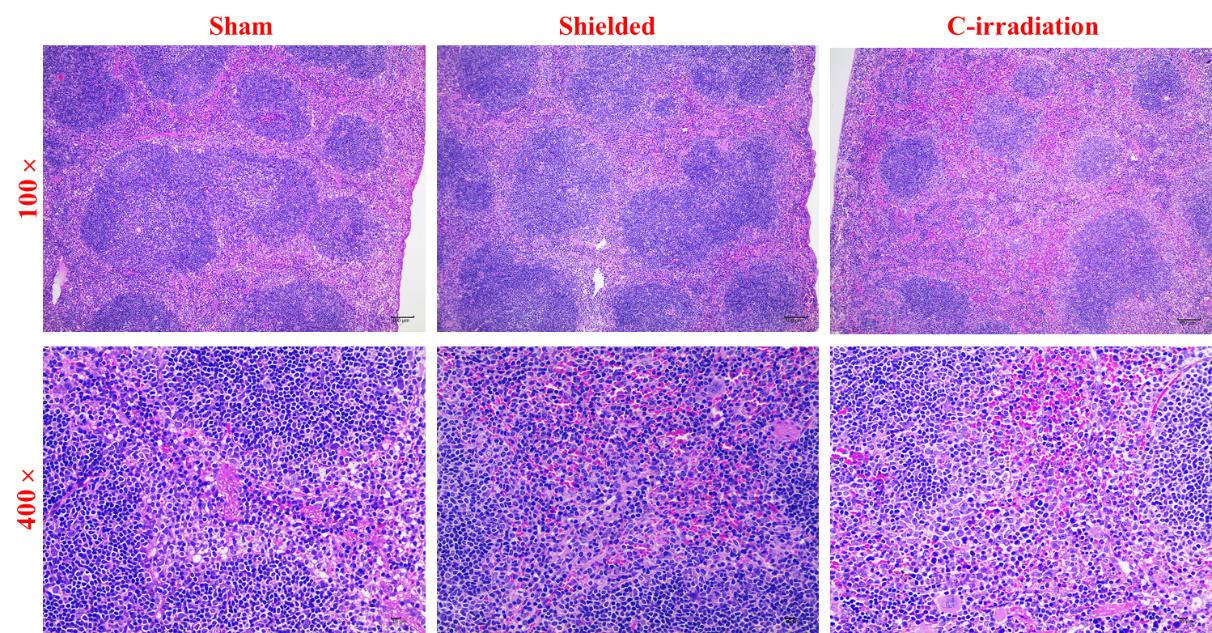


**Figure S4.** The abscopal effects of C-irradiation on the spleen of mice, HE staining, bar = 10 μm for 100 × magnification and 100 μm for 400 × magnification.


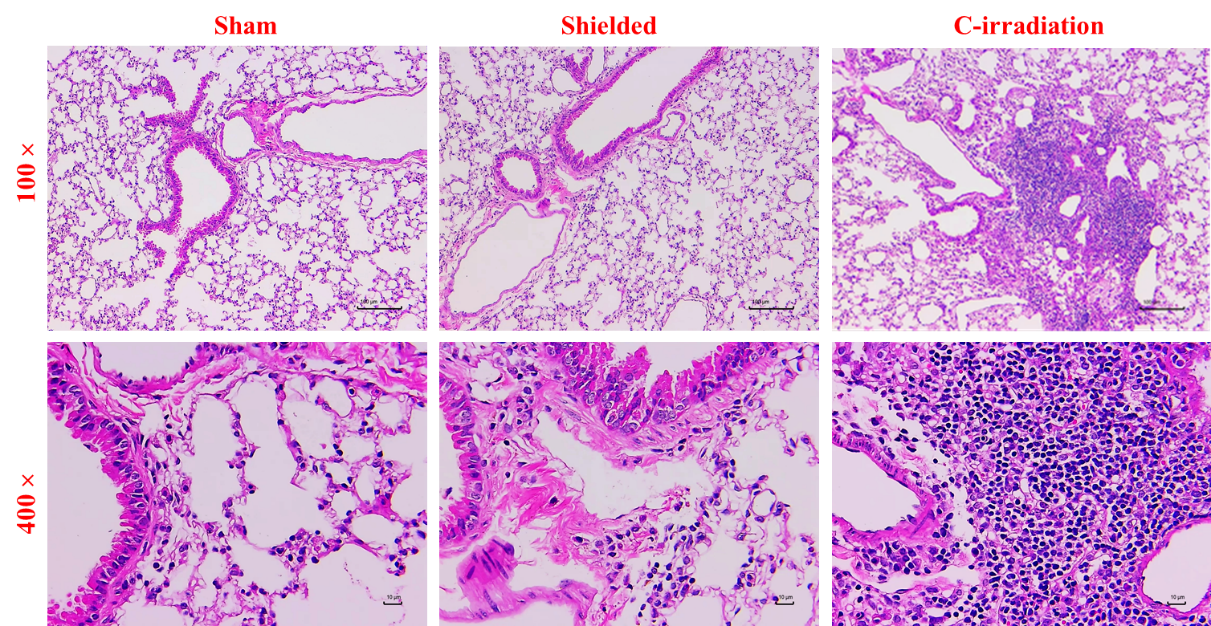


**Figure S5.** The abscopal effects of C-irradiation on the lung of mice, HE staining, bar = 10 μm for 100 × magnification and 100 μm for 400 × magnification.


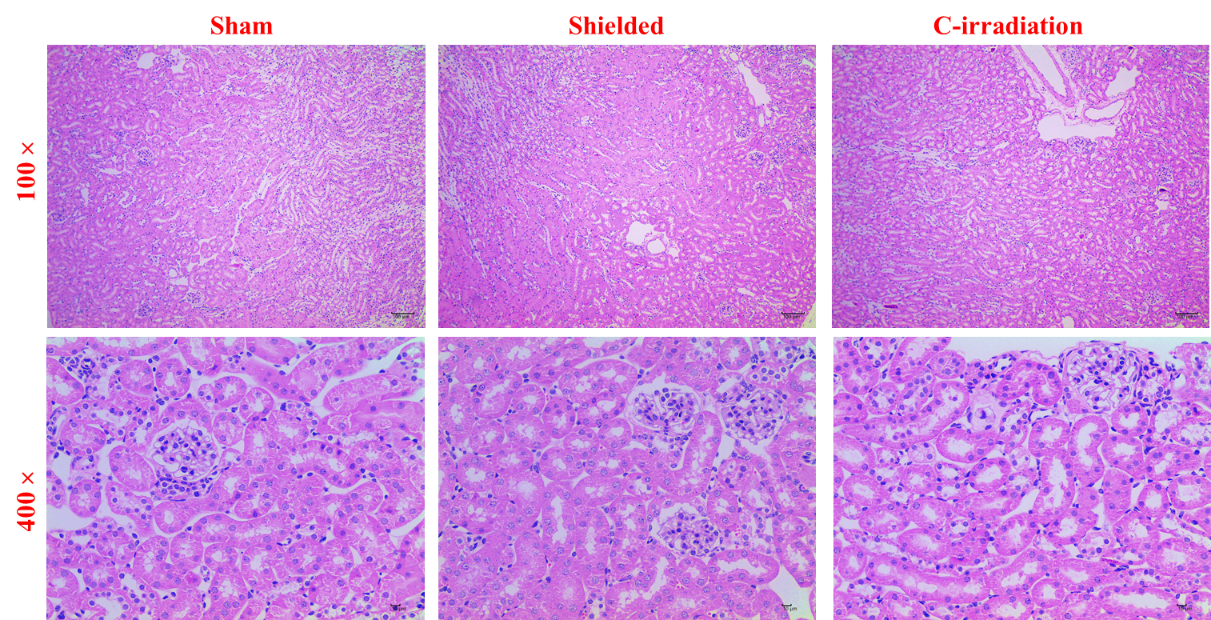


**Figure S6.** The abscopal effects of C-irradiation on the kidney of mice, HE staining, bar = 10 μm for 100 × magnification and 100 μm for 400 × magnification.


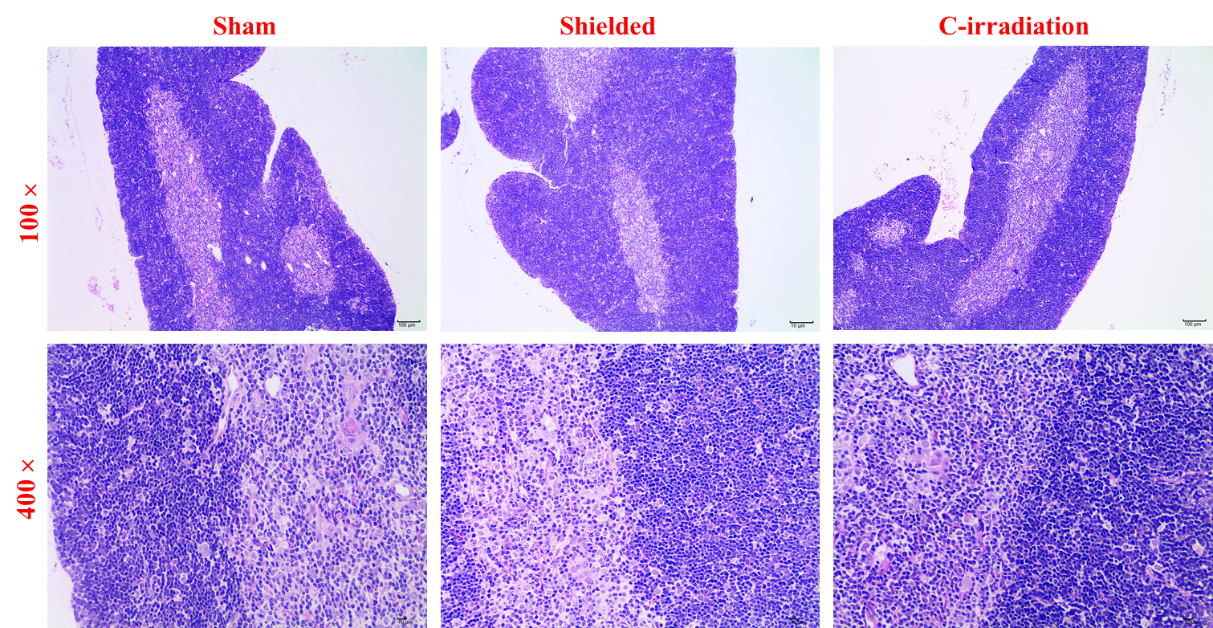


**Figure S7.** The abscopal effects of C-irradiation on the thymus of mice, HE staining, bar = 10 μm for 100 × magnification and 100 μm for 400 × magnification.
